# The tumour marker Fascin is strongly induced by Tax of HTLV-1 through NF-κB signals in T lymphocytes

**DOI:** 10.1186/1742-4690-8-S1-A186

**Published:** 2011-06-06

**Authors:** Andrea K Kress, Martina Kalmer, Aileen G Rowan, Ralph Grassmann, Bernhard Fleckenstein

**Affiliations:** 1 Institute of Clinical and Molecular Virology, Friedrich-Alexander-Universität Erlangen-Nürnberg, Erlangen, Germany; 2 Department of Immunology, Wright-Fleming Institute, Imperial College, London, W2 1PG, UK

## 

In search of novel biomarkers of HTLV-1- transformed cells with relevance to oncogenesis, we identified the tumour marker and actin-bundling protein Fascin (FSCN1) to be specifically and strongly upregulated in both HTLV-1-transformed and ATLL-patient-derived CD4+ T cells. Fascin is important for migration and metastasis in various types of cancer. Here we report that a direct link can exist between a single viral oncoprotein and Fascin expression, as the viral oncoprotein Tax of HTLV-1 was sufficient to induce high levels of Fascin. In contrast, Tax-2 of HTLV-2 could not induce Fascin. Expression of Fascin was also detectable in primary CD4+ T cells of ATLL-patients after they expressed Tax spontaneously. Immunofluorescence analysis revealed that Fascin could colocalize with Actin in the cytoplasm and at the membrane of HTLV-1-transformed cells. We found a novel mode of transcriptional regulation of Fascin by showing the importance of NF-κB signals for Tax-mediated induction of Fascin in T cells. Chemical and dominant-negative inhibition of the NF-κB pathway as well as a NF-κB-deficient Tax-mutant led to a strong decrease of Fascin mRNA and protein in Tax-transfected Jurkat cells. Additionally, in HTLV-1-/Tax-transformed cells Fascin transcripts were strongly reduced after chemical inhibition of IκB-kinase. Knockdown of Fascin by lentiviral transduction of shRNA decreased the invasive capacity of ATLL-derived cells into extracellular matrix. Thus, we have clearly shown that the tumour marker Fascin can be induced by the viral Tax oncoprotein through NF-κB signals. Our data suggest that Fascin up-regulation by Tax contributes to the development of HTLV-1-associated pathogenesis.

